# Evaluation of Ceftriaxone utilization in internal medicine wards of general hospitals in Addis Ababa, Ethiopia: a comparative retrospective study

**DOI:** 10.1186/s40545-015-0047-1

**Published:** 2015-11-09

**Authors:** Tariku Shimels, Arebu I Bilal, Anwar Mulugeta

**Affiliations:** Federal Police Commission Health Services Directorate, Addis Ababa, Ethiopia; Departement of Pharmaceutics and Social Pharmacy, School of Pharmacy, College of Health Sciences, Addis Ababa University, P.O. Box: 1176, Addis Ababa, Ethiopia; Department of Pharmacology, College of Health Sciences, School of Medicine, Addis Ababa University, Addis Ababa, Ethiopia

**Keywords:** Ceftriaxone, Drug use evaluation, Ethiopia, General hospitals, Medicine wards, Retrospective study

## Abstract

**Objectives:**

The irrational use of reserved antimicrobials, such as ceftriaxone, is one of the global public health issues particularly to low income countries like Ethiopia, leading to high costs of treatment or therapeutic failure. The purpose of the present study, thus, is to evaluate the appropriateness of ceftriaxone utilization in the medicine wards of general hospitals in Addis Ababa, with reference to the standard treatment guideline of Ethiopia for general hospitals.

**Methods:**

An institution based retrospective cross sectional study design was conducted in the internal medicine wards of Hayat and Zewditu Memorial hospital from 20 January to 20 February, 2014. Medication records of all patients who were admitted and prescribed with ceftriaxone during the previous one year to the study period were evaluated in reference to the Ethiopian Standard Treatment Guideline (STG 2010) for general hospitals.

**Results:**

The proportion of patients who received ceftriaxone was 59.3 % and 49 % in the public & the private hospital, respectively. Pneumonia, meningitis and sepsis were the common diagnoses in which ceftriaxone was prescribed in both hospitals. Maintenance fluids were the top ranked co-prescribed drugs in either hospital. Only 48.9 % in the public hospital and 44.6 % of records in the private hospital showed overall ceftriaxone use compliance to the guideline. Days of hospital stay was associated with appropriateness of ceftriaxone therapy.

**Conclusions:**

Even though ceftriaxone is one of the most commonly prescribed drugs in both hospitals, appropriateness of its use, according to the Ethiopian standard treatment guideline, was less than expected. This was so mainly from its indication and duration of therapy. Increasing the duration of hospital stay, however, showed to improve the appropriateness of ceftriaxone utilization.

## Introduction

Antimicrobials have played a remarkable role in public health through decreasing of morbidity and mortality [1]. The 19^th^ and 20^th^ are a particular centuries when longevity and health of populations progressed as the result of substantial achievements in controlling infectious diseases [1, 2]. This, however, was not without any challenge. Anti-microbial resistance (AMR) due to the over use of the reserved antibiotics is one of the threatening global issues of public health [3–5]. Many patients suffer due to harms arising from AMR because the infections caused by viruses, bacteria, fungi, protozoa and helminthes are no longer susceptible to the commonly available antibiotics. As a result, several stakeholders at every corner are striving to stimulate policies and actions in the healthcare system. As emphasized in a report by WHO, the Possible consequences that may arise include loss of productivity (loss in income, diminished worker productivity, time spent by family) and increased cost of diagnostics and treatment (consultation, infrastructure, screening, cost of equipment and drugs). In the same instance, while both the health and economic consequences of AMR are sought to be considerable and costly, it is difficult to quantify precisely as the available data are usually incomplete in many countries. The additional human burdens associated with it (pain, change in daily activities, psychosocial costs) are also inevitably significant [6].

Low-income countries are also the least to regulate drug distribution and utilization quality. The drug regulatory bodies have the responsibility of ensuring that drug products dispensed to patients will provide the necessary outcome and will not harm lies within the healthcare system of a country. Quality assurance, therefore, begins with licensing and monitoring of such healthcare providers at all levels. While this is particularly important for proper use of antimicrobials which are in high demand in the countries with high infectious disease burden including Ethiopia, there are findings showing that the practice is generally poor leading to the emergence of AMR pathogens [5, 7–10]. Therefore, conducting drug use evaluation studies in country like Ethiopia will have a profound importance in assessing the extent of the problem and to respond to it in a timely manner.

Detailed concerns in drug use evaluation may include inappropriate drug selection, incorrect dose, prescribing drugs that cause ADRs or drug interactions, and the use of expensive drugs when cheaper ones would be possible. In recognizing this, WHO recommended the alternative way of evaluating medicines utilization pattern in a more rigorous manner in health facilities to identify inappropriate uses and to promote rational utilization of medicines through drug and therapeutic committees [11].

A survey conducted in all regions of Ethiopia showed that more than 70 % have had one or more antimicrobials prescribed in a range of 1-6 (and with an average of 1.5) [12]. Ceftriaxone, as one of the most frequently prescribed 3^rd^ generation cephalosporin, with a reputable broad spectrum activity, relative low cost and availability in many developing countries is highly prone for misutilization [13, 14]. Few studies have evaluated the utilization of the drug in public hospitals of Ethiopia. Studies conducted in Ethiopia (Black lion and Police hospital, Ayder referral hospital, and Dessie referal hospital), revealed that there were inappropriate use of ceftriaxone [15–17]. The purpose of the present study, thus, is to evaluate the appropriateness of ceftriaxone utilization in the medicine wards of general hospitals in Addis Ababa, with reference to the standard treatment guideline of Ethiopia for general hospitals.

## Methods

### Study setting

The study was conducted in the medicine wards of Hayat and Zewditu memorial hospital. Hayat hospital is a privately owned general hospital located in Bole sub-city, Addis Ababa. It is established in 1999 and has a total number of 70 beds with an average of 346 daily patient flows. Hayat medical college is an affiliate of the hospital that teaches in medicine and Bachelor of Science in Nursing. The hospital also provides medical services on payment to the general population including referrals from nearby health institutions and regions.

Zewditu memorial hospital is also a public hospital administered under health bureau of the city government of Addis Ababa. Currently, the hospital has a total of 174 beds and provides referral medical services for patients coming from all sub cities and remote areas out of Addis Ababa. Established in 1932, Zewditu memorial hospital is also affiliated with the oldest Nursing school. On average, the hospital serves nearly 378 patients per day.

### Study design

A retrospective cross sectional study design was conducted to evaluate the utilization of ceftriaxone in Hayat and Zewuditu memorial hospital from 20 January, 2014 to 20 February, 2014**.**

### Inclusion and exclusion criteria

This study has included all the inpatients that have been admitted to Hayat and Zewditu memorial hospital medicine wards and stayed for more than 24 h. The patients who had received at least one course of any dose of ceftriaxone treatment within the twelve months prior to the start of the data collection month were considered. Medication records that had incomplete information in main variables like Socio-demographic, drug related traits, and diagnosis type were excluded.

### Data abstraction format

Data collection format was developed based on the WHO criteria [11] for Drug Use Evaluation, the manual for Drug and Therapeutics Committee training course by MSH and WHO [18] as well as the drug use evaluation Criteria prepared by the American Society of Health System Pharmacists [19]. The Ethiopian Standard Treatment Guideline of 2010 for general hospitals [20] was used to validate the tool for ceftriaxone in terms of indication, dosage, frequency and duration of treatment. Ceftriaxone treatment outcome measures were not considered since the study took a retrospective data.

### Data collection procedure

Before the start of data collection, the codes (card number, age, gender, and name) and treatment dates of case records for all inpatients attended the medicine wards were obtained from the patient registration documents. The charts of patients treated with ceftriaxone were then traced back to the records office of the hospitals using the card numbers obtained from the inpatient registration documents. The traced patient records in the registration book were recorded chronologically on to a separate form to include all the patient medication records. Data was collected for information on assessment, sex, age, dose, frequency of administration, days of hospital stay, number of co-morbid conditions, number and type of co-prescribed drugs with ceftriaxone and duration of antibiotic therapy.

### Ethical consideration

The study was approved by the School of Pharmacy Research and Ethics committee, Addis Ababa University. An approval and cooperation letter was also obtained from the Addis Ababa health bureau written to each hospital. The confidentiality of the data collected was maintained and all patient direct and quasi-identifiers (as name, address, phone number, card number, employer organization and name of relative…) were de-identified.

### Data analysis

The coded data were checked for completeness and entered in to Statistical package for social sciences (SPSS) version 20 for analysis. The WHO drug use evaluation criteria set namely indication; dose, frequency and duration were used as major measurements against the standard treatment guideline of Ethiopia. Inappropriate use of ceftriaxone in the hospitals was determined by comparing the observed ceftriaxone prescribing in the chart records to the recommendations in the guideline.

A descriptive analysis was employed and results are presented in tables and a figure. A binary logistic regression was used to determine the association between selected independent variables (age, sex, days of hospital stay, number of co morbidity and number of drugs co-prescribed) and the appropriate use of ceftriaxone. Statistical significance was considered at 95 % confidence interval.

## Results

A total of 477 patient medication records (charts) from both hospitals were included for analysis. Among these, 376 were from the public hospital and 101 were from the private hospital. The proportion of ceftriaxone prescription out of all medical ward admissions was 59 % and 49 % in the public and the private hospital respectively. From the 376 patients in the public hospital, 204 (54.3 %) were female. Of the 101 patients in the private hospital, 68 (67.3 %) were male. Mean age of patients in the public hospital was 44.6 years (±SD; 13.4) while in the private hospital it was 49.8 years (±SD; 21.0). Majority of treatment duration was in the range of 2–7 days accounting for 76.6 % and 89.1 % of the public and private hospital, respectively (Table 1). The median length of hospital stay was 11 and 6 days in the public and private hospital, respectively.

**Table 1:** Age, gender distribution and length of stay of patients in the medicine wards of general hospitals in Addis Ababa, January, 2014 (*n* = 477)

Hospital					
	Public			Private	
		N	%	N	%
Age (Years)					
	14-65	329	87.5	73	72.3
	>65	47	12.5	28	27.7
Gender					
	Male	172	45.7	68	67.3
	Female	204	54.3	33	32.7
Length of stay					
	0-7 days	110	29.2	76	75.2
	8-14 days	133	35.4	17	16.8
	>14 days	133	35.4	8	8.0
Total		376	100.0	101	100.0

### Common indications of ceftriaxone in the hospitals

Ceftriaxone was commonly utilized for pneumonia, meningitis, sepsis and non-surgical prophylaxis in both hospitals. It was also prescribed for hepatic disorders, UTI, cellulitis, and diabetic foot ulcer, in the public hospital. Similarly in the private hospital, ceftriaxone was indicated commonly for hepatic disorders, Urinary Tract Infection (UTI), cellulitis, diabetic foot ulcer, and Acute Gastro Enteritis (AGE) (Table 2).

**Table 2:** The most common diseases for which ceftriaxone was prescribed in the medicine wards of general hospitals in AA, February, 2014 (*n* = 477)

	Public hospital		Private hospital	
	Total	Comply to the guideline	Total	Comply to the guideline
Diagnoses	N	N (%)	N	N (%)
Pneumonia	152	105(69.1)	24	13(54.2)
Meningitis	62	47(75.8)	13	11(84.6)
Sepsis	50	22(44)	13	11(84.6)
Non-surgical prophylaxis	32	0	7	0
Hepatic disorders	17	0	5	0
UTI	16	0	8	2(25)
Cellulitis	12	3(25)	1	1(100)
Diabetic foot ulcer	9	6(66.7)	4	2(50)
Abscess	8	0		-
COPD	4	0		-
Wound	4	0		-
AGE	3	0	5	0
Pelvic inflammatory diseases	2	1(100)	5	1(20)
STDs	2	0		-
Bronchitis	1	0	5	0
Typhoid fever	1	0	6	4(66.7)
Upper GI bleeding	1	0		-
PUD	-	-	1	0
Tonsillitis	-	-	3	0
Epididymitis	-	-	1	0
Total	376	184(48.9)	101	45(44.6)

### Frequently co-administered drugs with ceftriaxone in the hospitals

Among the drugs co-administered with ceftriaxone, maintenance fluids took the lead in both hospitals, the proportion being twofold in the private hospital compared to the public hospital (72.28 % vs. 35.37 %). Metronidazole and anti-TBs in the public hospital whereas diclofenac and metoclopramide in the private hospital was the other common co-prescribed drugs (Table 3).

**Table 3:** The most frequently co-administered drugs with ceftriaxone in the medicine wards of general hospitals in AA, February, 2014 (*n* = 477)

Public hospital (*N* = 376)			Private hospital (*N* = 101)		
Drug	Frequency	Percent	Drug	Frequency	Percent
Maintenance fluids	133	35.37	Maintenance fluids	73	72.28
Metronidazole	124	32.98	Diclofenac	36	35.64
Anti-TBs	93	24.73	Methochlopramide	22	21.78
Cimetidine	77	20.48	Anti-Diabetics	18	17.82
Azithromycin	68	18.09	Tramadol	17	16.83
Furosemide	66	17.55	Vitamin B complex	16	15.84
Dexamethasone	62	16.49	Metronidazole	13	12.87
Cotrimoxazole	55	14.63	Quinine	9	8.91
Tramadol	52	13.83	Anti-TBs	8	7.92
Heparin	50	13.30	Furosemide	8	7.92
Spironolactone	47	12.50	Esomeprazole	7	6.93

### Overall STG compliance of ceftriaxone therapy

One hundred ninety two of the 376 patients’ charts (48.9 %) in the public hospital were found to be complying for an overall evaluation of indication, dose, and frequency of administration and duration of ceftriaxone treatment according to the 2010 Standard Treatment Guideline (STG) of Ethiopia for general hospitals. Using a similar procedure, 45 of the 101 patients (44.6 %) who were administered with ceftriaxone in the private hospital were found to comply with the guideline.

**Fig. 1:**
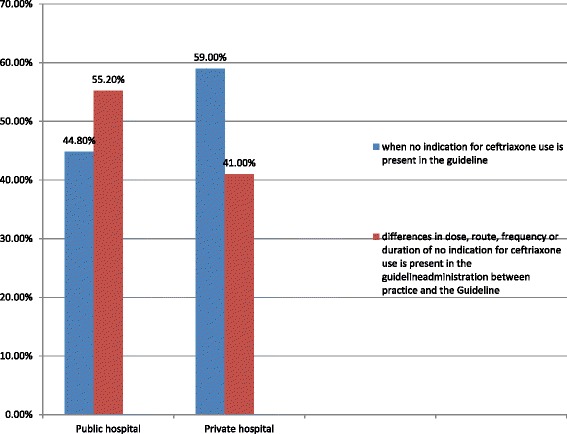
Proportion of Overall compliance use of ceftriaxone among medicine wards of general hospitals in AA, February, 2014 (*n* = 250)

Of the total 192 patient charts identified as inappropriate in the public hospital, 86 (44.8 %) was wrong indication of ceftriaxone as per the guideline whilst in 106 (55.2 %) cases were noted to show differences in dose, route, frequency or duration of administration between practice and the Guideline (Fig. 1). Wrong uses of ceftriaxone in the assessments of hepatic disorders, non-surgical prophylaxis, abscess, Chronic Obstructive Pulmonary Disease (COPD), wound, gastroenteritis, Bronchitis and upper gastro intestinal bleeding for which the guideline recommends no indication of the drug was noticed in the study.

In addition, deviation in duration of treatments for pneumonia, sepsis, meningitis, UTI, cellulitis and diabetic foot ulcer were noted (Table 2). Other than duration, appropriate doses were not mentioned in the treatment of pneumonia, meningitis, and sepsis.

### Factors associated with STG compliant use of ceftriaxone

From the results of the binary logistic regression, it was found that the likelihood of appropriate ceftriaxone use increased with days of hospital stay in both the public and the private hospital (AOR = 6.00; 95 % CI [3.40–10.80]), (AOR = 3.30; 95 % CI, [1.00–9.12]). This association works even when days of hospital stay increase beyond 14 days in the public hospital (AOR = 5.58; 95 % CI, [3.10–10.00]) while it failed to show statistical significance in the private hospital (AOR = 3.50; 95 % CI, [0.75–16.58]). Other variables namely, age, sex, co morbidity and presences of co administered drugs were not associated with appropriateness (Table 4).

**Table 4:** Factors associated with appropriateness of ceftriaxone utilization in the medicine wards of general hospitals in AA; February 2014 (*n* = 477)

				Crude		Adjusted	
	Factors	N	%	OR	95 % CI	OR	95 % CI
Public hospital	Age						
	14–65 Yrs.	329	87.5	1.34	0.72–2.49	0.80	0.40–1.50
	>65 Yrs.	47	12.5	1.00	1.00	1.00	1.00
	Sex						
	Male	172	45.7	0.91	0.61–1.37	0.95	0.60–1.5
	Female	204	54.3	1.00	1.00	1.00	1.00
	Days of hospital stays						
	0–7 days	110	29.25	1.00	1.00	1.00	1.00
	8–14 days	133	35.40	5.90	3.30–10.50	6.00	3.40–10.80
	>14 days	133	35.40	5.710	3.20–10.15	5.58	3.10–10.00
	Co morbidity						
	0	60	16.0	1.00	1.00	1.00	1.00
	> = 1	316	84.0	1.68	0.95-2.95	1.60	0.89–3.00
	Co-prescribed						
	0	6	1.6	1.00	1.00	1.00	1.00
	> = 1	370	98.4	0.95	0.19–4.807	1.10	0.20–6.00
Private hospital	Age						
	14–65 Yrs.	73	72.3	0.64	0.34–1.94	1.60	0.60–4.00
	>65 Yrs.	28	27.7	1.00	1.00	1.00	1.00
	Sex						
	Male	68	67.3	1.40	0.59–3.34	1.20	0.50–3.10
	Female	33	32.7	1.00	1.00	1.00	1.00
	Days of hospital stay						
	0–7 days	76	75.2	1.00	1.00	1.00	1.00
	8–14 days	17	16.8	3.00	1.00–9.11	3.00	1.00–9.12
	>14 days	8	8.0	3.61	0.80–16.37	3.50	0.75–16.58
	Comorbidity						
	0	37	36.6	1.00	1.00	1.00	1.00
	> = 1	64	63.4	1.50	0.65–3.55	1.23	0.48–3.20
	Co-prescribed						
	0	2	2.0	1.00.	1.00	1.00	1.00
	>1	99	98.0	0.63	0.03–10.30	0.38	0.02–6.73

## Discussion

The extent of ceftriaxone utilization in the present study was comparably different among the public and the private hospital. A higher proportion (59.3 %) of the patients admitted to the medicine ward of the public hospital have taken at least a single dose of the drug compared to the private hospital (49 %) in the previous one year. A higher figure was reported by a prospective evaluation in a teaching hospital medicine ward of India accounted for 72 % [21]. This higher figure could result due to the shorter period of evaluation considered (5 months). However, a lower result (34 %) was, also, reported by another study in an educational hospital of Tehran [22]. A prospective cross sectional study in the tertiary care hospital of India [23] has shown an equivalent proportion as the private hospital in this evaluation with an amount of 48.5 %. This is probably because; the proportion represents the percentage of patients in the medicine ward who had been administered with only ceftriaxone despite the utilization of other cephalosporin. The variation in the extent of ceftriaxone utilization among the public and the private hospital might be contributed to the availability and use of different generations of cephalosporin in the private hospital leading to lower ceftriaxone consumption.

A higher proportion (48.9 %) of the charts in the public hospital showed appropriate prescribing of ceftriaxone relative to the private hospital (44.6 %). Though the two hospitals were comparable in many aspects, this difference might have been in effect by the availability of DTC/DIC, hospital specific drug lists as well as the STG of Ethiopia in the public hospital while all were absent in the private hospital. These figures are lower as compared to studies conducted inside the country [15, 17]. The reasons for the above difference could be the shorter evaluation period affecting the overall compliance level. Lower figures were also reported by similar studies conducted in Ethiopia (Ayder referral hospital), Iran, Australia, and Thailand [16, 22, 24, 25].

Majority (55.5 %) of the noncompliance to STG in the public hospital were related to differences in dose, route, frequency and duration of administration between practice and the guideline. At the same time, prescribing of ceftriaxone for the treatments of hepatic disorders, non-surgical prophylaxis and abscess which are not mentioned in the guideline was also observed. Higher proportions, (58.9 %), of noncompliance in the private hospital were indication of ceftriaxone for non-surgical prophylaxis, bronchitis, hepatic disorders and tonsillitis. Noncompliance to the STG was observed in the duration of treatment for pneumonia, pelvic inflammatory disease, typhoid fever and meningitis. Inappropriate use of ceftriaxone for bronchitis was reported in another study conducted inside the country [16]. The use of ceftriaxone for prophylaxis in high risk non-surgical patients of the public hospital was very high (32 %) compared to the case in the private hospital (7 %).

The most common inappropriate use of ceftriaxone was deviation from the recommended treatment duration. This is complemented by most of the drug use evaluation studies in Ethiopia which indicated duration as a common deviation from the guideline, majorly in the managements of pneumonia, meningitis and sepsis [15–17]. A similar study in Thailand has also revealed that duration and indication of ceftriaxone therapy were main components of inappropriate use [25].

From the results of the binary logistic regression, it was found that the appropriate use of ceftriaxone increased with days of hospital stay in both the public and the private hospital. Most of the inappropriate medication uses in the medical patients arise due to short duration of therapy than is recommended in the guideline. Short durations, in turn and partly, arise when the patients are not able to afford for admission (bed) costs, medical and non-medical expenses for most reasons. Though the median length of hospital stay in the public hospital (11 days) was longer than that in the private hospital (6 days) probably due to costs mentioned above, it is evident that increased length of stay would alleviate deviations from proper duration of therapy.

Pneumonia, meningitis and sepsis were common cases in which ceftriaxone was prescribed in both hospitals. Similar findings in Ethiopia were also reported that pneumonia, meningitis and sepsis are the top cases which ceftriaxone was indicated [15–17]. This implies that these diseases are prevalent and known to consume the drug in most admissions of Ethiopian hospitals.

Among the drugs co-prescribed with ceftriaxone, maintenance fluids ranked at the top in both hospitals in which the proportion being a twofold in the private hospital (72.28 % vs. 35.37 %). Metronidazole (32.98 %) and anti-TBs (24.73 %) accounted the next high percentage of co-prescribed drugs in the public hospital whereas diclofenac (35.64 %) and metoclopramide (21.78 %) occupied the same order in the private hospital. The overall pattern in both hospitals showed that infectious diseases were the major co morbidities followed by chronic disease (Diabetes, hypertension, hyperlipidemia and stroke). Intravenous (IV) incompatibility could be a possible threat in the study areas as maintenance fluids involving ringer lactate were noted to be co-prescribed.

## Limitations of the study

The evaluation was relied, merely, on the medication records of patients/charts for which the drug use by the patient or administration by health care professional might have, pragmatically, been different.

## Conclusion

Ceftriaxone is one of the most commonly prescribed drugs for treatment and prevention of a disease in both hospitals. However, the use of the drug in a significant number of cases was not in accordance to the Ethiopian standard treatment guideline. Indication and duration of ceftriaxone treatment were the aspects in which noncompliance were frequently noted. Of factors considered, duration of hospital stay was found to be predictive for appropriate use of this drug. Having strict antibiotic policy and complete adherence to national and international treatment guidelines may alleviate the problem.

## Abbreviations

ADR: Adverse drug reaction
AGE: Acute gastro enteritis
AMR: Anti-microbial resistance
ASHP: American Society of Hospital Pharmacists
COPD: Chronic obstructive pulmonary disease
DIC: Drug Information Center
DTC: Drug and Therapeutics Committee
IV: Intravenous
STG: Standard treatment guideline
UTI: Urinary tract infections
